# Oxidative Stress in Parasitic Diseases—Reactive Oxygen Species as Mediators of Interactions between the Host and the Parasites

**DOI:** 10.3390/antiox13010038

**Published:** 2023-12-23

**Authors:** Marta Pawłowska, Celestyna Mila-Kierzenkowska, Jan Szczegielniak, Alina Woźniak

**Affiliations:** 1 Department of Medical Biology and Biochemistry, Faculty of Medicine, Ludwik Rydygier Collegium Medicum in Bydgoszcz, Nicolaus Copernicus University in Toruń, 85-092 Bydgoszcz, Poland; celestyna_mila@cm.umk.pl (C.M.-K.); al1103@cm.umk.pl (A.W.); 2 Physiotherapy Department, Faculty of Physical Education and Physiotherapy, Opole University of Technology, 45-758 Opole, Poland; j.szczegielniak@po.edu.pl; 3 Ministry of Internal Affairs and Administration’s Specialist Hospital of St. John Paul II, 48-340 Glucholazy, Poland

**Keywords:** oxidative stress, parasites, host, parasitic diseases, reactive oxygen species

## Abstract

Oxidative stress plays a significant role in the development and course of parasitic infections, both in the attacked host organism and the parasite organism struggling to survive. The host uses large amounts of reactive oxygen species (ROS), mainly superoxide anion (O_2_^•−^) and hydrogen peroxide (H_2_O_2_), to fight the developing parasitic disease. On the other hand, the parasite develops the most effective defense mechanisms and resistance to the effects of ROS and strives to survive in the host organism it has colonized, using the resources and living environment available for its development and causing the host’s weakening. The paper reviews the literature on the role of oxidative stress in parasitic diseases, which are the most critical epidemiological problem worldwide. The most common parasitosis in the world is malaria, with 300–500 million new cases and about 1 million deaths reported annually. In Europe and Poland, the essential problem is intestinal parasites. Due to a parasitic infection, the concentration of antioxidants in the host decreases, and the concentration of products of cellular components oxidation increases. In response to the increased number of reactive oxygen species attacking it, the parasites have developed effective defense mechanisms, including primarily the action of antioxidant enzymes, especially superoxide dismutase and nicotinamide adenine dinucleotide phosphate hydrogen (NADPH)-dependent complexes glutathione and thioredoxin.

## 1. Introduction

In recent years, there has been a notable surge in scholarly attention toward parasitic diseases. These ailments represent a prevalent public health concern, particularly among individuals embarking on journeys to regions characterized by a tropical climate [1,2]. A noteworthy problem is the potential for these infections to give rise to dangerous complications. Of significance is the protracted subclinical or mildly symptomatic persistence of parasitic diseases, which can extend for several months [3].

The involvement of oxidative stress (OS) is a notable determinant in the progression and dynamics of parasitic infections, manifesting its influence within the afflicted host organism and the parasite itself as it grapples with persisting [4,5,6]. Simultaneously, the parasite employs intricate defense mechanisms and cultivates resilience against the impacts of reactive oxygen species (ROS), aiming to sustain its survival within the colonized host organism [5,6]. This adaptation entails strategically utilizing available resources and the host’s living habitat to facilitate its growth, concurrently inducing a state of debilitation in the host organism. ROS play an essential role in defense against invasion by parasites and microorganisms [7]. The host uses the harmful effects of ROS on the parasite’s cells to destroy it. Phagocytic cells, which include granulocytes, monocytes, and macrophages, in response to the attack of an undesirable invader in the organism, react with increased (even several dozen times) oxygen consumption, the so-called oxygen shock [8]. This is to produce and release large amounts of superoxide anion (O_2_^•−^) and hydrogen peroxide (H_2_O_2_) to fight bacterial, viral, and parasitic infections [5].

Maintaining homeostasis is critical for each living organism [9]. It is necessary for the proper functioning of metabolism, the occurrence of all life activities essential for survival, and the proper functioning of the immune system in defense against infection. The oxidant–antioxidant balance should remain undisturbed so the organism can perform all these essential functions. In the case of the host, the oxidative balance is vital for maintaining, as far as possible, the protective effect of barriers against invasion by undesirable individuals and effective combat in the event of an existing parasite infection [9]. Also, in the case of the parasite, maintaining the oxidative balance is essential to benefit from the interaction for as long as possible and defend itself against oxidative stress generated in the host’s organism. The parasite weakens the host organism and facilitates colonization by causing oxidative stress [5].

This study aims to present the role of ROS as a mediator of interactions between the host and various parasites attacking it and the role of OS in parasitic diseases. In addition, this paper would like to emphasize the beneficial effects of ROS in defending the host organism against parasite invasion. By analyzing selected literature on experimental studies that raise the issue of the role of OS in the course of parasitic diseases, the authors will try to answer questions about the role of oxidative stress in the system of infections with selected parasites, the role of ROS in the host organism’s fight against the invading and exploiting parasite, and the parasite’s response to shock.

## 2. Methods

We thoroughly reviewed the literature, utilizing databases such as Web of Science, PubMed, Science Direct, and Google Scholar to gather information on oxidative stress in parasitic infections. The following keywords were used in data retrieval: (“oxidative stress and parasites”, “oxidative stress and host”, “oxidative stress and protozoa”, “oxidative stress and helminths”, “oxidative stress and parasitic infections”); (“oxidant-antioxidant balance and parasites”, “oxidant-antioxidant balance and host”, “oxidant-antioxidant balance and protozoa”, “oxidant-antioxidant balance and helminths”, “oxidant-antioxidant balance and parasitic infections”). Data collection was inorganic, except for abstracts or conference reports, where full text was unavailable.

## 3. Oxidant–Antioxidant Balance

Oxygen constitutes about one-quarter of the Earth’s mass, and over 50% of the elements in the Earth’s crust are oxygen atoms (mainly in the form of oxides) [10]. Therefore, it is a ubiquitous element on our planet. O_2_ is undoubtedly essential for the survival of aerobic organisms. It enables aerobic organisms to obtain significantly more significant amounts of energy in a shorter time in the process of respiration than can be observed in the case of anaerobes [10]. However, this has inevitable consequences because oxygen, reacting with organic compounds, oxidizes them and reduces itself [11]. During complete reduction, four electrons and four protons are added to the oxygen molecule, whose primary state is the triplet state, forming two water molecules (O_2_ + 4 H^+^ + 4 e^−^ → 2H_2_O) [11]. The problem is that the reduction of O_2_ is not always four-electron, which results in the formation of free radicals, i.e., atoms or molecules with an unpaired electron in their valence shell [12].

### 3.1. Reactive Oxygen Species Formation

**Figure 1:**
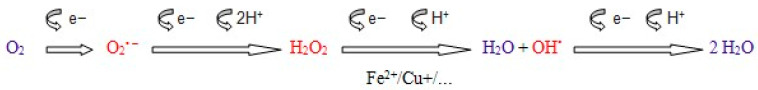
Oxygen reduction leads to the formation of reactive oxygen species.

Free radicals are more reactive than triplet oxygen because they strive to pair a free electron and form a permanent chemical bond [11]. As a result of the one-electron reduction of oxygen, which takes place during the mitochondrial respiratory chain, a superoxide anion radical (O_2_^•−^) is formed [12]. After adding another electron to O_2_^•−^, we can obtain hydrogen peroxide (H_2_O_2_), both as a result of a spontaneous reaction and in a dismutation reaction catalyzed by the enzyme–superoxide dismutase (SOD) [13]. H_2_O_2_ in vivo is produced primarily from O_2_^•−^ transformations taking place in mitochondria, but it quickly diffuses through cell membranes and in the presence of metal ions of transition groups, significantly often Fe^2+^, perhaps in the Fenton reaction (H_2_O_2_ + Fe^2+^ → OH^•^ + Fe^3+^ + OH^−^) lead to the formation of a hydroxyl radical (OH^•^) (see Figure 1). OH^•^ is the product of three electrons added to the oxygen molecule [14]. It is the most reactive compound in biological systems [15]. In addition to Fe^2+^, Cu^+^, Co^2+^, chromium, and nickel can catalyze the Fenton reaction [14].

All oxygen reduction and excitation products are more reactive than oxygen in the ground state. Therefore, they are called reactive oxygen species [16]. The term ROS includes free oxygen radicals, such as superoxide anion radical, hydroxyl radical, and nitric oxide (NO^•^), and non-radical oxygen combinations, including mainly hydrogen peroxide, hypochlorous acid (HOCl), singlet oxygen (^1^O_2_) and ozone (O_3_), as well as other molecules created as a result of metabolic reactions [16].

ROS may be formed in the human organism due to cell metabolic reactions or external physical factors in the organism, such as ionizing radiation, ultrasound, and ultraviolet radiation [17]. Apart from the inside of the organism, we also deal with ROS, which are found in the external environment, both in water and the air. The most important cellular source of ROS is the mitochondrial respiratory chain. It generates nearly 90% of the ROS produced in the organism, even though cytochrome oxidase reduces oxygen by four electrons during the production of ATP [18]. However, the flow of electrons through the respiratory chain is partially tight. In a competitive reaction, reduced coenzyme NADH dehydrogenase and ubiquinone forms enter into a one-electron reaction with oxygen, forming O_2_^•−^. This applies to 1–4% of the oxygen mitochondria consume during respiration [19].

Peroxisomes are also the site of O_2_^•−^ production, produced here by xanthine oxidase and a short electron transport chain consisting of NADH reductase and cytochrome b5. NADH reductase acts as an electron acceptor inside the peroxisome [20]. It then passes them on to cytochrome b5, which reduces unidentified physiological substrates. However, cytochrome b5 can also transfer them to oxygen molecules, resulting in the formation of O_2_^•−^ [21]. Peroxisomes are also the primary source of H_2_O_2_ in the cell, but the release of large amounts of it into the cytoplasm is prevented by catalase in these organelles [20].

Other sources of ROS in the human organism are oxidation of reduced forms of low molecular weight cells, e.g., proteins containing thiol groups -SH, oxidation of xenobiotics (drugs, food ingredients), and oxidation of respiratory proteins: hemoglobin, present in erythrocytes, and myoglobin, present in muscles, whose groups heme cells contain Fe^2+^, which creates an excellent opportunity for the Fenton reaction to occur [22].

Many years of experiments conducted on various cells have shown the adverse effects of ROS on cellular structures. By disrupting cell function, ROS can ultimately lead to cell death [23]. ROS’s targeted action affects all cell component classes, including low molecular weight compounds, proteins, lipids, and nucleic acids [24]. The greatest threat to our organism comes from the action of the hydroxyl radical because it is the most reactive of all ROS, and we do not have an enzyme specific for it in the organism, as is the case with H_2_O_2_ or O_2_^•−^ [25].

The most thoroughly studied biological free radical chain process is lipid peroxidation, i.e., the formation of peroxides through free radical oxidation of unsaturated fatty acids or other lipids [26]. Most often, polyunsaturated heavy acid residues that are part of phospholipids are subjected to such oxidation because phospholipids are the primary building component of cell membranes [27]. The initiator of lipid peroxidation may be, among others, OH^•^, O_3_, HOCl or NO^•^, as well as nitrogen dioxide (NO_2_^•^) and sulfur dioxide (SO_2_^•^). However, this reaction is not initiated by O_2_^•−^ [28]. The end products of this reaction are modified lipid molecules, including phospholipid dimers and oxo- or hydroxy fatty acids [26]. These products undergo further transformations, leading to the breakdown of polyunsaturated fatty acid residues and the formation of smaller fragments, among which the best known is malondialdehyde (MDA), used as a marker of lipid peroxidation [29].

ROS also cause oxidative damage to proteins, leading to the loss of their biological activity [24]. This happens due to modifying amino acid residues of protein prosthetic groups and fragmentation or aggregation of protein molecules. Some modifications, such as oxidation of thiol groups of proteins -SH, may occur under the influence of O_2_^•−^ or H_2_O_2_, but the most common mediator of protein oxidation is OH^•^ [30].

Among the low molecular weight compounds damaged by ROS, the most important are glutathione, ascorbate, and uric acid. By being oxidized in reaction with ROS, they protect the organism against more severe consequences, which will be discussed in the section on antioxidant defense [24].

Nucleic acids are more stable than other cell components, and H_2_O_2_ and O_2_^•−^ do not damage them. However, like other organic molecules, DNA is also attacked and damaged by OH^•^ and ^1^O_2_ [31]. Free radical DNA damage includes modifications of nitrogenous bases, formation of DNA-protein cross-links, breaks in one or two DNA strands, and DNA methylation disorders. The accumulation of nucleic acids altered this way may result in gene expression, cell death, and/or carcinogenesis changes [32]. Hypomethylation leads to changes in gene expression, which may induce the expression of proto-oncogenes. Mutations may arise as a result of improper pairing of modified bases during DNA replication or as a result of repairing damage [32].

### 3.2. Antioxidant Defense Mechanisms

Due to the many unfavorable effects of ROS, cells of living organisms have developed mechanisms in the course of evolution that allow them to eliminate or reduce the harmful effects of ROS [33]. This function is performed by antioxidants, i.e., substances that, in small concentrations, protect cell components against oxidation or significantly delay this process. Neutralization of ROS results from the interaction of many reactions conditioned by several antioxidants involved in the organism’s defense. The antioxidant defense system has three lines of protection [33].

The first line of defense is specific antioxidant enzymes, which aim to prevent ROS formation, especially OH^•^, and to prevent the already formed ROS from reacting with biologically active compounds [34]. In human organisms, some enzymes selectively disproportionate H_2_O_2_ (catalase, glutathione peroxidase) and an enzyme that selectively decomposes O_2_^•−^ (superoxide dismutase). These enzymes belong to the so-called antioxidant triad. However, humans do not have an enzyme specific for OH^•^ [35]. The action of the mentioned enzymatic triad is to prevent the formation of this form of ROS, and non-enzymatic OH^•^ scavengers are responsible for its neutralization.

Superoxide dismutase (SOD) is an enzyme that catalyzes the dismutation reaction of superoxide anion [36]. There are three types of this enzyme in humans: Copper-zinc superoxide dismutase (Cu/ZnSOD, SOD-1) occurs mainly in the cytosol and in smaller amounts in the intermembrane space of mitochondria. Its activity was also found in the cell nucleus, peroxisomes, and lysosomes [37]. Among organs, the liver and testicles have the highest dismutase activity, and erythrocytes have the most increased enzyme activity among cells [38]. Manganese superoxide dismutase (MnSOD, SOD-2) is located in mitochondria and peroxisomes and occurs in all organism cells, with the highest concentration documented in heart, brain, liver, and kidney cells. It is a homotetramer, each subunit of which has one manganese atom in the active center, which serves both a catalytic and enzyme-stabilizing function [36]. The increase in SOD-2 activity protects cells against oxidative stress and influences cancer development. An increase in MnSOD levels inhibits growth factors in cancer cells in vivo and in vitro [39]. Extracellular superoxide dismutase (EC-SOD, SOD-3) mainly occurs in the extracellular matrix and on the cell surface. It is present in tissues, blood plasma, and extracellular fluids such as lymph, synovial fluid, cerebrospinal fluid, semen, and others. Human SOD-3 is most significant in blood vessels, lungs, and placenta [40].

The following important antioxidant enzyme is catalase (CAT), which breaks down hydrogen peroxide. This enzyme is a homotetramer, and each subunit has a heme system with a central iron atom [41]. Disproportionation of H_2_O_2_ with catalase involves two steps. In the first stage, the heme iron of catalase is oxidized by hydrogen peroxide, contributing to the formation of a porphyrin radical, which in the second stage is reduced by another molecule of H_2_O_2_, resulting in the shape of water and molecular oxygen [42].

Another enzyme of the antioxidant triad is glutathione peroxidase (GPx), whose task is to reduce H_2_O_2_ and organic peroxides with the participation of reduced glutathione. It is a tetramer containing selenocysteine in the active center, which enables glutathione oxidation without releasing the free glutathione thiol radical [41]. There are five isoforms of this enzyme: cytosolic (cGPx, GPx-1), plasma (pGPx, GPx-3), nuclear (snGPx), gastrointestinal (giGPx, GP-2), and phospholipid hydroperoxide peroxidase (phGPx, GPx-4). Present in mitochondria and cytoplasm [43]. Increased cGPx activity is observed in neutrophils, suggesting its anti-inflammatory effect [44].

The cooperation of all enzymes of the antioxidant triad is essential [41]. The necessary protective function of SOD is beyond doubt, but one of the following two enzymes must cooperate with it. GPx has a greater affinity for H_2_O_2_, suggesting it is more critical in most physiological situations where H_2_O_2_ concentrations are low. However, the other can compensate for lacking one of these enzymes [45].

The second line of defense is the so-called “scavengers” of ROS, designed to direct free radical reactions towards termination [46]. This group includes low-molecular-weight antioxidants of exogenous or endogenous origin, inhibiting oxidative processes by reacting with oxidizing factors–preventive antioxidants, or with oxidation intermediate products, usually ROS—interventional antioxidants [47]. Their reactions with ROS are less specific than the previously mentioned enzymatic reactions, which makes them more universal guardians of the organism and can perform many different functions. These compounds can react with O_2_^•−^ or H_2_O_2_, inhibiting the formation of OH^•^, but they also respond with OH^•^ if produced. They function as the second line of defense by directing free radical reactions to termination pathways [46]. The essential hydrophilic antioxidants for the human organism are glutathione and vitamin C. However, this group also includes several other compounds: flavonoids (e.g., resveratrol), uric acid, creatinine, cysteine, carnosine, and neopterin [48]. The most critical hydrophobic antioxidants responsible for protecting cell membranes include vitamin E, carotenoids, bilirubin, and biliverdin, reduced coenzyme Q and estrone and estradiol derivatives, and vitamin D_3_ [47,49,50].

The third and last line of defense is based on repairing or removing molecules damaged by ROS. The components of this line of defense mainly protect DNA in the organism [51]. DNA repair enzymes usually repair DNA damage. Ligases can repair single-strand breaks. Damaged bases are removed by cutting them out with glycosylase and then removing this fragment, which is performed by a specific endonuclease. Next, the missing element is synthesized again based on the complementary strand by the polymerase and integrated with the rest of the strand via ligase. However, if the damage is so significant that the cell cannot repair it, the SOS system is activated, making a quick but imprecise repair so DNA replication can occur. This inaccurate DNA repair may result in its mutation but protects the cell against apoptosis [52].

All lines of defense form an interconnected and mutually complementary network of defense mechanisms and antioxidant compounds. To maintain the organism’s homeostasis, it is vital to maintain a balance between the production of ROS and their elimination in enzymatic and non-enzymatic neutralization and scavenging reactions due to the action of exogenous antioxidants. Disturbance of this balance is called oxidative stress [53].

### 3.3. Oxidative Stress

OS occurs in many situations due to exposure of the organism or its cells to additional sources of ROS due to increased endogenous ROS production or antioxidant deficiency. As mentioned earlier, the adverse effects of ROS underlie many disorders and diseases [53]. They cause damage to all classes of molecular components of cells and may ultimately lead to the cell’s death or even the entire organism. The metabolic consequences of oxidative stress in cells include lowering ATP levels [54]. Due to the inactivation of glyceraldehyde-3-phosphoaldehyde dehydrogenase, glycolysis is inhibited. In addition, the catabolism of adenine nucleotides is increased, and the supply of reduced forms of nicotine adenine dinucleotides is diminished due to their use for glutathione reduction and DNA damage repair [55]. Another effect of OS in cells is decreased total glutathione concentration. Glutathione conjugated with other compounds is removed by active transport outside the cell, which contributes to the consumption of ATP and lower glutathione concentration in the cell [43]. Moreover, there is an increase in the concentration of calcium in the cytoplasm caused by the inactivation of the calcium pump, as well as an increase in membrane permeability as a consequence of its depolarization and DNA damage due to the action of OH^•^ and the activation of nucleases [56,57].

## 4. Oxidative Stress in the Host

During parasitic infections, the host’s immune system often responds by generating increased levels of ROS as a defense mechanism. ROS, such as superoxide radicals and hydrogen peroxide, are crucial in attacking and eliminating the parasites [58]. However, excessive and prolonged production of ROS can lead to OS, causing damage to host tissues and contributing to the overall pathology of the infection [59,60]. OS in the host of parasitic infection can damage biomolecules, including lipids, proteins, and nucleic acids. ROS can initiate lipid peroxidation, protein oxidation, and DNA damage, compromising cells’ structural and functional integrity. This damage may exacerbate the host’s susceptibility to infection and contribute to developing chronic conditions [61]. The enzymatic antioxidant defense protects host cells, shielding them from abundant free radicals resulting from parasitic infections [6].

Some parasites have developed sophisticated strategies to manipulate the host’s immune response, including modulation of OS [62]. Parasites may produce antioxidant enzymes or molecules that help evade the host’s ROS attack. This modulation can create an imbalance in the host’s antioxidant defense system, promoting oxidative stress and impairing the effectiveness of the immune response [5]. Understanding these mechanisms is crucial for developing targeted interventions to enhance the host’s ability to combat parasitic infections.

### 4.1. Protozoa Infections

*Plasmodium* parasites that cause malaria in humans are considered one of the most essential parasitological and epidemiological problems in the world because over 40% of the world’s population lives in areas where malaria is endemic [63,64]. On a global scale, two species are most often responsible for infections: *P. vivax* and *P. falciparum*, causing 95% of detected malaria cases [65]. If infected with a parasite of this species, untreated invasion may lead to death. Malaria is a severe systemic disease characterized by complications such as multi-organ failure [66]. It has been found that OS plays a vital role in the development of malaria [67,68]. During the development of the *Plasmodium* spp. in the host organism, the production of ROS by phagocytic cells increases, and the “oxygen burst” they generate is aimed at combating the developing *Plasmodium* spp. ROS detoxification is also a big challenge for erythrocytes infected with *Plasmodium* spp. [69]. Due to the high metabolic rate of rapidly multiplying parasites, large amounts of toxic, active redox reaction products are generated. The degradation of hemoglobin of host blood cells by *Plasmodium* spp. is also of fundamental importance for generating oxidative stress—degradation in the acidic environment of the vacuole results in the production of toxic-free heme and ROS [70]. The presence of malaria parasites in erythrocytes causes changes that may shorten the survival time of these cells, which does not allow the full development of the intracellular parasite. Toxic metabolites lead to a reduced ability of erythrocytes to protect against ROS due to increased lipid peroxidation inside blood cells and to increased susceptibility of erythrocytes to damage and also cause disturbances in the structural integrity of erythrocytes due to the lack of some essential metabolites [71]. Exposure of erythrocytes to OS is associated with disorders of their membrane, which leads to a shortened survival time of red blood cells and an increased removal of them from the bloodstream [70]. Changes in the erythrocyte membrane are caused by changes in its fluidity, probably resulting from lipid peroxidation and protein cross-linking caused by increased oxidative stress. In addition, erythropoiesis is also impaired, so the production of blood cells is also reduced. These changes in the production and functioning of erythrocytes result in anemia during malaria [71].

Malaria infection may be associated with oxidative damage and a reduction in α-tocopherol reserves in the erythrocyte membrane. This suggests that local antioxidant depletion may contribute to erythrocyte loss in severe malaria [72]. The presence of α-tocopherol in the erythrocyte membrane is a better indicator of the organism’s exposure to ROS than in the plasma [73]. OS mediates tissue damage during *P. vivax* infection [74]. The concentration of MDA and antioxidants, including antioxidant enzymes, may be helpful to direct markers of OS during malaria [75,76].

During *P. falciparum* infections, host red blood cells are exposed to oxidation and OS, while the parasite is effectively equipped with antioxidants to protect it from oxidative damage. The parasite has enormous potential for de novo GSH synthesis and reduction of glutathione disulfide (GSSG) [77]. The portion of GSSG actively shed by the parasite is reduced to GSH in the host cells, whose own GSH synthesis is paralyzed. Glutathione reductase and thioredoxin reductase are essential enzymes for maintaining appropriate intracellular redox homeostasis in the parasite’s organism and, therefore, for its adaptation and survival in increased OS [78]. Many studies focused on OS in *Plasmodium* spp. and other protozoan infections were conducted. The results of some studies are presented in Table 1.

*Toxoplasma gondii* is a globally distributed obligate intracellular protozoan pathogen responsible for causing toxoplasmosis [6]. *T. gondii* can infect virtually any nucleated cell in warm-blooded animals and is present in approximately one-third of the global human population [79]. In the acute phase of toxoplasmosis, ROS production is significant, leading to the induction of OS in the tissues of infected animals. This heightened oxidative response is the host’s defense mechanism against the infection [6].

Protozoan parasites from the *Trypanosomatidae* family induce debilitating diseases in numerous regions worldwide, particularly in developing countries characterized by tropical and subtropical climates. Within this family, *Trypanosoma cruzi* is the causative agent of Chagas disease [80,81]. In response to *T. cruzi* infection, activated macrophages exhibit cytotoxic effects on parasites by producing ROS and reactive nitrogen species (RNS). The multimeric complex NADPH oxidase (NOX2) utilizes NADPH as a substrate, reducing O_2_ to generate peroxide (O_2_^•−^), which subsequently undergoes dismutation into the stable and diffusible pro-oxidant H_2_O_2_ [81]. According to existing literature, the persistent presence of parasites in the heart is likely influenced by the limited ability of macrophages to generate a robust ROS/NO response and the parasites’ capacity to scavenge oxidants. This scenario contributes to prolonged parasite persistence and mitochondrial oxidative stress within the heart. The resulting cellular oxidative damage stimulates macrophage activation and the induction of chronic inflammatory stress in Chagas disease [81].

Contracting the protozoan parasite *Leishmania* spp. has the potential to result in the onset of leishmaniasis, a significant yet often overlooked disease [82]. Host macrophages exhibit their most potent anti-*Leishmania* response by generating ROS and reactive nitrogen species (RNS). This highly controlled process is designed to eliminate invading pathogens without causing harm to the host cell. Activated in part by phagocytosis, this mechanism engages various enzymes. NOX2 and inducible nitric oxide synthase (iNOS) are the primary contributors to reactive species production in macrophages, generating superoxide (O_2_^−^) and nitric oxide (NO), respectively [62].

**Table 1:** Summary of selected research studies on oxidative stress in protozoan infections.

Study Group	Parasite	Results	Ref.
72 patients with malaria, 40 healthy subjects (control)	*Plasmodium falciparum*	decreased vit. A, vit. C and vit. E levels, decreased GSH activity, increased MDA level in malaria patients	[83]
63 patients with malaria, 67 healthy subjects (control)	*Plasmodium* spp.	decreased SOD and GSH activities and increased MDA level in malaria patients	[76]
17 patients with complicated malaria, 51 patients with uncomplicated malaria, 15 healthy subjects (control)	*Plasmodium* spp.	decreased vit. C level, increased MDA level in malaria patients	[84]
551 patients with malaria, 221 healthy subjects (control)	*Plasmodium* spp.	decreased GST, SOD, and CAT activities, increased MDA level in malaria patients	[85]
60 patients with malaria, 30 healthy subjects (control)	*Plasmodium falciparum*	decreased vit. C level, decreased SOD activity in malaria patients	[86]
80 patients with malaria, 80 healthy subjects (control)	*Plasmodium falciparum*	decreased vit. A, vit. C, vit. E levels, decreased GSH activity, increased MDA level in malaria patients	[87]
20 infected patients, 20 patients with diarrhea, 30 healthy subjects (control)	*Entamoeba histolytica*	decreased GSH, CAT, and SOD activities, increased MDA level in infected patients	[88]
50 infected patients (giardiasis), 32 infected patients (toxoplasmosis), 40 healthy subjects (control)	*Giardia duodenalis*, *Toxoplasma gondii*	decreased GSH activity, increased MDA and NO levels in infected patients	[89]
44 seropositive pregnant women, 40 healthy pregnant women (control)	*Toxoplasma gondii*	decreased vit. C and vit. E levels, decreased GSH, SOD, GPx, and CAT activities, increased MDA level in seropositive women	[90]
50 seropositive patients, 30 healthy subjects (control)	*Toxoplasma gondii*	decreased GSH activity, increased MDA level in seropositive patients	[91]
50 seropositive patients, 50 healthy subjects (control)	*Toxoplasma gondii*	increased MDA level in seropositive patients	[92]
65 infected patients, 50 healthy subjects (control)	*Leishmania donovani*	decreased CAT activity, increased MDA level in infected patients	[93]
28 infected patients, 10 healthy subjects (control)	*Leishmania braziliensis*	increased NO production in monocytes of infected patients	[94]

CAT—catalase, GPx—glutathione peroxidase, GSH—glutathione, GST—glutathione transferase, MDA—malondialdehyde, NO—nitric oxide, SOD—superoxide dismutase.

### 4.2. Helminth Infections

The most common parasitic infections both in Poland and in the world concern infections with intestinal parasites [95]. These invasions mainly affect children, especially those living in larger groups. Each species is a cosmopolitan parasite, but *Enterobius vermicularis* infection predominates in temperate climates, while *Trichuris trichiura* and *Ascaris lumbricoides* infections are favored by hot and humid climates. In addition to weather, an important factor determining the occurrence of these infections is the level of sanitation in a specific area [96,97]. Among flatworms, the most common infections are caused by tapeworms of the genus *Taenia*: *T. saginata* and *T. solium*. The most significant number of cases of unarmed tapeworm infection are recorded in sanitarily neglected areas and areas where the custom of eating raw beef is common [98,99].

Intestinal parasite infections may initially be asymptomatic or cause non-specific symptoms, mainly in the gastrointestinal tract. Intestinal problems, such as abdominal pain, nausea, vomiting, diarrhea, constipation, and loss of appetite or weight, primarily manifest them. In children, stunted growth and weakness may also occur [98,100]. In addition to these symptoms of intestinal parasite infections, these parasites also play a role in inducing OS in the host organism, contributing to the associated adverse consequences [101]. Table 2 shows the results of some studies on OS conducted on patients with helminth infections.

The OS induced during helminth infections can influence the host’s immune response. It may result in altered immune cell functions, affecting the balance between pro-inflammatory and anti-inflammatory signals. This modulation of the immune response contributes to the complex interplay between the host and the helminth, influencing the course and outcome of the infection [102,103].

Within the intestine, phagocytic cells, as integral components of the mucosal immune response, can generate ROS and RNS. Additionally, the epithelium and microbiota can contribute to the production of ROS and RNS in this context [101]. The increase in LPO and GSH concentrations accompanying infection and the changes in the activity of antioxidant enzymes increasing with the duration of illness indicate a decrease in the effectiveness of protecting the host’s gastrointestinal tract against OS during the parasitic infection [101,104]. While many studies are currently available in which oxidative stress parameters were determined in patients with protozoan infection, few studies are performed to determine oxidative stress parameters in patients with helminth infection.

**Table 2:** Summary of selected research studies on oxidative stress in helminth infections.

Study Group	Parasite	Results	Ref.
40 infected patients (enterobiasis), 46 infected patients (echinococcosis), 40 healthy subjects (control)	*Enterobius vermicularis*, *Echinococcus granulosus*	decreased GSH activity, increased MDA and NO levels in infected patients	[89]
20 infected patients, 10 healthy subjects (control)	*Fasciola hepatica*	decreased GPx and SOD activities, increased CAT activity, increased MDA level in infected patients	[105]
140 infected patients, 140 healthy subjects (control)	*Fasciola hepatica*	increased GPx, CAT, and SOD activities, increased MDA level in infected patients	[106]
12 infected rats, 12 uninfected rats (control)	*Fasciola hepatica*	increased NOX and TBARS levels in infected rats	[107]
29 infected dogs, 16 uninfected dogs (control)	*Ancylostoma* spp.	increased TAC level in infected dogs	[108]
22 infected cattle (fasciolosis), 30 infected cattle (cysticercosis), 40 healthy cattle (control)	*Fasciola hepatica*, *Cysticersus bovis*	decreased GSH, GST, SOD, and CAT activities, increased MDA levels in infected cattle	[109]

CAT—catalase, GPx—glutathione peroxidase, GSH—glutathione, GST—glutathione transferase, MDA—malondialdehyde, NO—nitric oxide, NOX—nicotinamide adenine dinucleotide phosphate oxidase, TAC—total antioxidant capacity, TBARS—thiobarbituric acid reactive substances, SOD—superoxide dismutase.

## 5. Oxidative Stress in the Parasite

Oxidative stress plays a crucial role in influencing the survival and proliferation of parasite organisms. When exposed to the host’s immune response or antiparasitic drugs, parasites often experience an increase in ROS production, leading to OS [110]. This oxidative assault can damage crucial biomolecules within the parasite, affecting its ability to evade the host immune system and reproduce [111]. Parasite organisms have developed intricate adaptive mechanisms to counteract OS and enhance their chances of survival within the host. These adaptations include expressing antioxidant enzymes and synthesizing small-molecule antioxidants. By neutralizing ROS and minimizing cellular damage, parasites can better navigate the hostile oxidative environment created by the host’s immune system [62].

Researchers are exploring compounds that selectively enhance OS within parasites, exploiting their weakened antioxidant defenses. This approach aims to tip the balance in favor of the host’s immune system, making it easier to eliminate the parasites. Targeting OS provides a promising avenue for developing more effective and selective antiparasitic treatments.

### 5.1. Protozoa Infections

As described previously, *P. falciparum* causes increased hemoglobin degradation in the infected person’s organism, disrupted erythrocyte production, and shortened survival time. These activities result in increased generation of ROS, which are expected to help fight the infection by harming the parasite’s organism. Parasites of the *Plasmodium* spp. are susceptible to OS during their development in the host’s erythrocytes, i.e., at the trophozoite stage [112]. However, the parasite does not give up so quickly, and to maintain the intracellular redox balance, it mainly uses the enzymatic activity of superoxide dismutase and the action of two NADPH-dependent complexes: the thioredoxin complex—based on thioredoxin reductase/thioredoxin—and the glutathione complex—based on glutathione reductase/reduced glutathione [113,114,115].

*P. falciparum* parasites have two superoxide dismutases. Fe-SOD, called SOD-1, found in the cytosol, appears to be transcribed and expressed during the parasite’s erythrocyte cycle. It also occurs in other species, including *P. vivax*, *P. malariae*, and *P. ovale* [112,116]. As SOD-1 is a cytosolic protein, it is unlikely to act on superoxide anions produced during the digestion of hemoglobin in the parasite’s vacuoles. Moreover, it cannot be ruled out that a large percentage of ROS generated in digesting vacuoles undergoes spontaneous dismutation under the influence of the acidic pH inside the vacuole [117,118]. *P. falciparum*’s second superoxide dismutase, SOD-2, is a protein found in mitochondria. The presence of mitochondrial SOD appears to be necessary for *Plasmodium* spp. having an active respiratory chain through which anions inevitably leak. SOD-2 is therefore responsible for their detoxification in the mitochondria, preventing disruption of metabolic functions and damage to nucleic acids, proteins, and membranes of cell organelles [119].

The thioredoxin system involves many biochemical reactions, including protecting cells against OS and cell proliferation and growth. It consists of enzymatic proteins: thioredoxin and thioredoxin reductase (TrxR), and NADPH, obtained mainly in the pentose phosphate cycle, is necessary for the proper course of the reaction [120]. Undoubtedly, the primary physiological function of the thioredoxin system is the detoxification of ROS, e.g., H_2_O_2_, S-nitrosoglutathione, and others. TrxR protects cells exposed to oxidative stress against apoptosis [121]. There are two isoforms of TrxR in *P. falciparum*: cytosolic (TrxR1) and mitochondrial (TrxR2) [122,123]. TrxR is necessary for subsequent stages of parasite development in the erythrocyte, which is why it is a target of antimalarial drugs. TrxR2, in particular, seems to be a promising target for new therapies due to its location in mitochondria [124]. It has been shown that inhibition of TrxR2 activity results in increased permeability of the mitochondrial cell membrane and cytochrome c release. In cells with increased TrxR production, resistance to drugs whose mechanism of action is based on increased ROS production has also been observed [125].

The glutathione complex also plays an essential role in developing *P. falciparum* in erythrocytes and maintaining its oxidative balance [112]. The redox state in the parasite organism living in the host’s blood cells largely depends on the reduction of glutathione and the circulation of GSSG and GSH. In *P. falciparum*, GSSG is mainly recycled by GR but can also be directly reduced by thioredoxin (Trx), plasmoredoxin, and dihydrolipoamide-dependent reactions catalyzed by glutaredoxin. GSSG reduction can be kept at high speed by the TrxR/Trx system [126]. At low TrxR and GR concentrations, the thioredoxin system is even more effective at reducing GSSG than GR [127]. Like thioredoxins, glutaredoxins are also common in animals and plants and are essential in lowering GSSG in proteins [128]. Unlike Trx, Grx is not limited to reducing specific substrates but reduces glutathione via a non-enzymatic pathway [129]. The thioredoxin system of *P. falciparum* can reduce GSSG more effectively than corresponding systems in other organisms, such as humans [112]. The thioredoxin and the glutathione complexes are essential in maintaining the intracellular oxidative balance. Still, the accepted concept is that both complexes act on the cellular homeostasis of the organism independently of each other [130]. A promising way to interrupt the redox homeostasis of *P. falciparum* is to block the reduction of NADP, on which both described complexes depend [131]. The sources of NADPH are glutamate dehydrogenase (GDH) and isocitrate dehydrogenase (IDH). However, the production of NADPH by GDH in *P. falciparum* is low compared to that in humans. NADPH in malaria parasites is supplied mainly in the first stage of the pentose phosphate cycle, catalyzed by a bifunctional enzyme with the activity of glucose-6-phosphate dehydrogenase and 6-phosphogluconolactonase [132,133].

Additionally, glutathione S-transferase (GST) functions in the glutathione system—an enzyme that catalyzes the reaction of attaching electrophiles and nucleophiles to glutathione. It occurs in many aerobic organisms, participating in cellular metabolism, detoxification, and excretion of endogenous and exogenous substances from the organism [134]. GST levels are likely regulated in vivo by ROS [135]. GST has been detected in all *Plasmodium* spp., and its activity is significantly increased in chloroquine-resistant parasites compared to susceptible parasites [136,137]. The *P. falciparum* parasite has one glutathione S-transferase. PfGST is also necessary for the parasite’s survival and its protection against OS, which is a potential target in the search for new antimalarial drugs [77]. *Plasmodium* spp. is sensitive to oxidative damage caused by the increased production of ROS in infected host blood cells. Therefore, blocking the antioxidant activity of PfGST is used in the design of new, effective antimalarial drugs [138].

Antioxidant systems are crucial in controlling OS and the survival of apicomplexan parasites within hosts. Parasites like *T. gondii* and *Babesia* spp. have evolved intricate antioxidant defense mechanisms, allowing them to thrive within host cells [6,139]. The challenge of OS extends beyond intracellular survival, as infection exposes parasites to the host’s immune system, which employs ROS to combat the infection. Apicomplexan parasites are particularly vulnerable to oxidative stress, making targeted interference with their redox homeostasis an excellent prospect for drug development [140].

As an obligatory intracellular parasite, *T. cruzi* must endure its own internally generated toxic metabolites, which emerge as by-products of its aerobic metabolism. Additionally, the organism must confront the oxidative burst originating from the host immune system. This burst encompasses the generation of O_2_^•−^ and various other ROS [80]. While ROS are traditionally associated with pathogen elimination during the oxidative burst, emerging evidence indicates that ROS production might be beneficial in the context of *T. cruzi* macrophage infection. OS triggers iron mobilization from the host’s intracellular storages, a crucial cellular factor necessary for the proliferation of amastigotes [80]. Goes et al. [141] also highlighted a paradoxical function of ROS, indicating that modified parasites exhibit enhanced replication within macrophages. However, the proliferation is markedly diminished when ROS are eliminated from the host cell. These findings propose a role for ROS as a signaling molecule, actively contributing to the growth of *T. cruzi* inside cells.

*Leishmania* spp. parasites actively navigate OS from their initial entry into the macrophage to their survival [62]. These parasites can either secrete or induce the production of arginase in macrophages. Arginase competes with inducible nitric oxide synthase (iNOS) for arginine, generating essential nutrients like l-ornithine for synthesizing polyamines and urea. Simultaneously, this process diminishes the production of parasitotoxic nitric oxide (NO). Additionally, the leishmanial metalloprotease gp63 plays a role in further inhibiting oxidative stress by interfering with macrophage signaling pathways, ultimately impeding the induction of NOX2 and iNOS [62]. Moreover, ROS contribute to the process of parasite differentiation. Alterations in intracellular iron levels activated a signaling pathway dependent on ROS, prompting the differentiation of infectious amastigotes into promastigotes [80].

### 5.2. Helminth Infections

Intestinal parasites, like the previously described protozoan parasites, also struggle to survive in the host’s organism and have evolved defense mechanisms to defend against oxidative stress generated in large amounts during an oxygen burst intended to enable the host to fight off the parasitic infection. Helminths employ a significant detoxification mechanism featuring diverse isoforms of glutathione S-transferases (GSTs). These multifunctional enzymes play a crucial role in cellular detoxification by facilitating the conjugation of endogenous and exogenous toxic chemicals to glutathione [101].

Filariae have both main types of SOD: MnSOD—contained in cell mitochondria—and CuZnSOD—located in the cytosol and peroxisomes [142]. The primary function of SOD is to protect parasite cell components from oxidative damage. However, its role in preserving the effector mechanisms of the immune system by limiting the production of ONOO^−^ and the subsequent production of OH^•^ by preventing O_2_^•−^ and NO^•^ reactions is also postulated [143]. GPx is also constantly synthesized and acts on the surface of parasite cells, protecting the cell membrane. The problem, however, lies in the deficient activity of this enzyme, which is probably caused by the low nucleophilicity of the cysteine-containing site compared to the selenocysteine present in the active area of human GPx [144]. The described means that parasites metabolize H_2_O_2_, which is still not a satisfactory response to the oxidative stress generated in the host organism.

Recently, other enzymes that can play an influential role in defending filariae against the oxidative effects of H_2_O_2_ have been searched for. It is indicated that the group of peroxiredoxins performs this function [145]. Peroxiredoxins are widely distributed in both prokaryotic and eukaryotic organisms. They are also common in protozoa and helminths [146,147].

Non-enzymatic antioxidant defense is equally important. Various low-molecular-weight substances, water-soluble, e.g., glutathione or ascorbate, and fat-soluble, e.g., α-tocopherol or ubiquinol, perform similar antioxidant functions [148]. However, no detailed studies have been conducted on the relative contribution of these substances to the antioxidant defense of filaria and parasitic helminths.

## 6. Conclusions

The host’s immune response to parasitic infections involves the generation of reactive oxygen species as a defense mechanism against parasites. While reactive oxygen species are crucial for combating parasites, their excessive and prolonged production can lead to oxidative stress, causing damage to host tissues and contributing to the overall pathology of the infection.

Parasites have evolved various defense mechanisms to counteract oxidative stress, including the production of antioxidant enzymes such as superoxide dismutase and glutathione peroxidase, as well as non-enzymatic antioxidants such as glutathione and α-tocopherol. In malaria infections caused by *Plasmodium* species, the parasite’s antioxidant defense mechanisms, such as the thioredoxin system and glutathione complex, are crucial for its survival in host erythrocytes. Understanding these mechanisms may help develop effective antimalarial drugs that target these specific pathways. Helminths also use similar antioxidant defense mechanisms, including superoxide dismutase and peroxiredoxins, to combat oxidative stress generated by the host immune response. However, lower levels of certain protective enzymes, such as glutathione peroxidase and catalase, make them susceptible to oxidative damage.

Oxidative stress induced by parasitic infections can lead to a variety of health complications in the host, including anemia, tissue damage, and impairment of the immune system, additionally exacerbating the effects of parasitic infections. Therefore, further research into the specific pathways and mechanisms involved in the parasite’s antioxidant defense systems is essential to develop effective treatments and interventions against parasitic infections and related complications caused by oxidative stress.

## Data Availability

The datasets presented in this study can be found in the online repositories. The name and accession number of the repository/repository can be found at https://www.ncbi.nlm.nih.gov/ (accessed on 28 November 2023), SRA: SRP358796, Bioproject accession: PRJNA804319.
